# Successful management of severe scalp avulsion injury: a case report and review of surgical interventions

**DOI:** 10.1093/jscr/rjae590

**Published:** 2024-09-18

**Authors:** Sujan Paudel, Prajjwol Luitel, Ishwor Thapaliya, Anup Thapa, Samit Sharma

**Affiliations:** Maharajgunj Medical Campus, Institute of Medicine, Tribhuvan University, Kathmandu 44600, Nepal; Maharajgunj Medical Campus, Institute of Medicine, Tribhuvan University, Kathmandu 44600, Nepal; Maharajgunj Medical Campus, Institute of Medicine, Tribhuvan University, Kathmandu 44600, Nepal; Department of Plastic Surgery, Tribhuvan University Teaching Hospital, Kathmandu 44600, Nepal; Department of Plastic Surgery, Tribhuvan University Teaching Hospital, Kathmandu 44600, Nepal

**Keywords:** avulsion, scalp avulsion, reconstructive surgery, skin grafting

## Abstract

Scalp avulsion injuries caused by machinery present substantial challenges necessitating urgent medical intervention. A 30-year-old female suffered near-complete scalp avulsion from entanglement in agricultural machinery and underwent surgical repair with a latissimus dorsi free flap and split-thickness skin graft. Free flap techniques offer reliable wound closure but may lead to cosmetic concerns. Safety measures in high-risk environments are crucial to prevent such incidents.

## Introduction

Degloving scalp injuries occurs when the skin and subcutaneous tissue of scalp are forcefully separated from the underlying fascia and muscles due to severe trauma [1, 2]. Scalp avulsions pose challenges due to their painful nature and complexity in treatment. Early patient stabilization, antibiotic prophylaxis, and surgical debridement are vital to remove necrotic tissue, reduce infection risk, and assess soft tissue loss [3]. Immediate scalp re-implantation with microsurgical anastomosis usually gives the best cosmetic results, but it may not always be possible due to the condition of the avulsed scalp [4]. Alternative reconstruction methods, such as free flaps, microvascular surgery, skin grafting have been documented [5]. Though microvascular reconstructions can offer favorable outcomes, they can complicate with flap necrosis, donor-site problems, scarring, prolonged surgery, and recovery time [6].

Following SCARE guidelines [7], we present a rare case of a 30-year-old female with scalp avulsion caused by her hair being caught in rotating agricultural machinery. This type of avulsion injury and its management outcomes are rarely documented in the literature.

## Case report

A 30-year-old female was referred to the Emergency Department with a history of a scalp avulsion 24 h back secondary to accidental drag by thresher machine. She denied loss of consciousness, abnormal body movement, weakness, vomiting, double vision, fever, dizziness, or bleeding from nose or ear.

**Figure 1:**
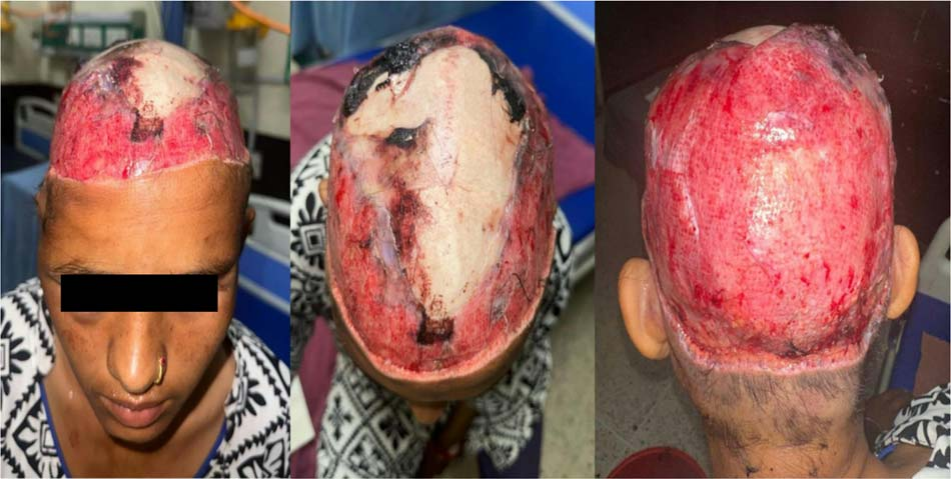
Near complete scalp avulsion at time of presentation.

She was alert, conscious, and vitals were stable. Examination revealed no fractures or bruises on her torso or extremities. A near-complete scalp degloving injury was identified, extending from the forehead around the entire circumference of the scalp to the upper nuchal line (Fig. 1). The avulsed scalp exhibited severe damage and fragmentation, making replantation unfeasible.

Laboratory results revealed normal blood counts. Renal, liver function tests, and extended focused assessment with sonography for trauma (e-FAST) scan were normal. Head computed tomography (CT) scans showed no signs of intracranial bleeding, skull fractures, or brain contusions. CT scans of the cervical spine, chest, and abdomen also showed no significant injuries.

**Figure 2:**
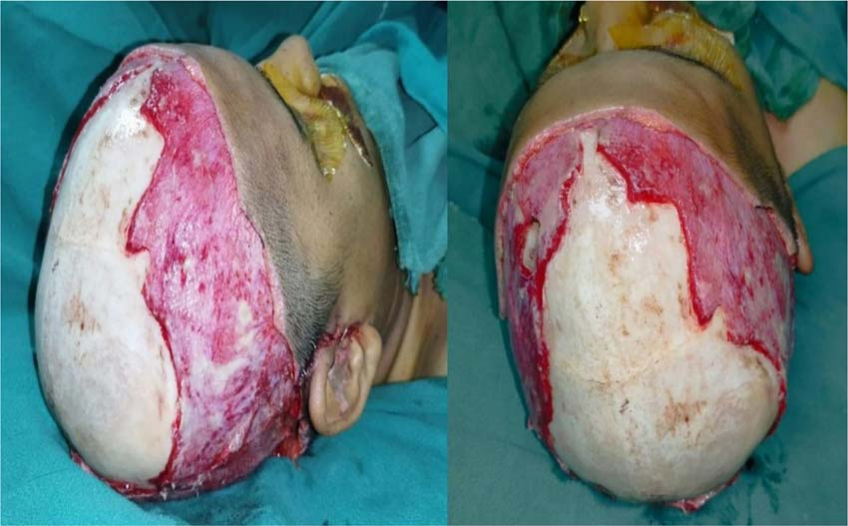
Exposed raw area following debridement.

**Figure 3:**
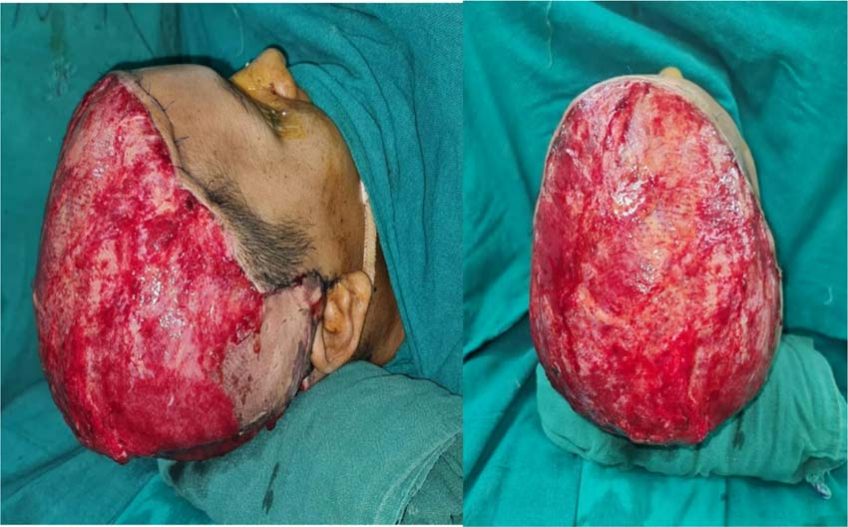
Coverage of scalp defect using latissimus dorsi free flap.

Intravenous fluids and antibiotics were initiated with application of topical antiseptic agents. The wound was irrigated, and debridement was done along with adequate hydration (Fig. 2). In the same setting, latissimus dorsi (14 cm) free flap was placed with anastomosis of the thoracodorsal vessel to the superficial temporal vessel (Fig. 3).

**Figure 4:**
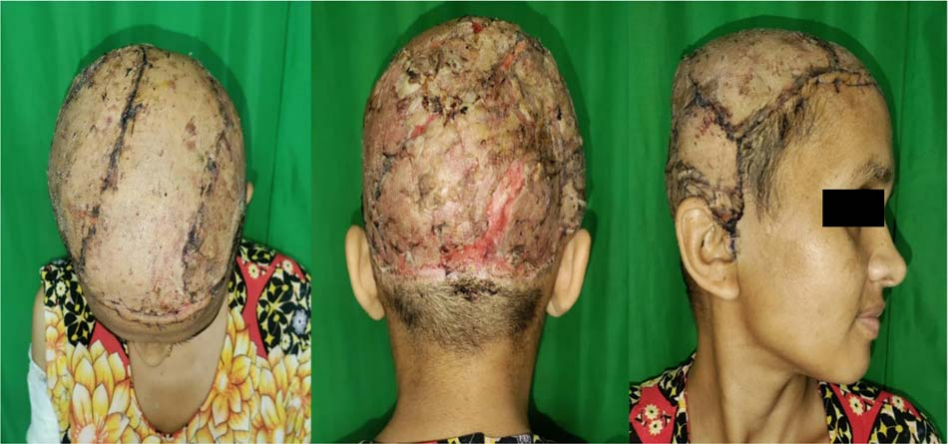
Successful uptake of STSG.

The patient tolerated the procedure well, requiring one pint of packed red blood cells transfusion due to falling hemoglobin levels. Postoperatively, she underwent daily dressing changes achieving satisfactory flap uptake. A week following flap placement, a split-thickness skin graft (STSG) was harvested from the thigh. The perioperative course was uneventful, and after satisfactory graft uptake, she was discharged after 2 weeks (Fig. 4). On follow-up at 12 months, she was satisfied with the outcome.

## Discussion

Total scalp avulsion is a rare occurrence, usually following industrial or high velocity road traffic accidents [8]. In our region, hair entrapment in agricultural machinery remains one of the most common causes, similar to our case in agricultural workplace without occupational safety gears [9]. Total scalp avulsion typically results from severe shearing forces applied obliquely to the hair-bearing scalp, often when long hair becomes caught in rotating agricultural machinery [4]. This traction causes separation between the galea aponeurotica and periosteum, involving muscles like frontalis, temporalis, and occipitalis in forming avulsion edges [10]. The forehead, portions of the ear, entire ear, eyebrows, and upper nasal skin are commonly affected in most avulsed scalp cases [4]. Scalp avulsion injuries may cause hypovolemic shock, potential intracranial, and cervical damage, requiring fluid resuscitation and head imaging [8]. However, our case did not demonstrate any such complications on evaluation.

The literature emphasizes size and location of the defect, history of radiation exposure, and hairline distortion for reconstruction of scalp defects [11]. Microsurgical replantation is the primary treatment for total scalp avulsion, depending on factors like scalp availability, ischemia time, and vessel viability [12]. Before microsurgery, scalp amputations were treated with composite or skin grafts, frequently resulting in alopecia due to follicle damage [9]. Free tissue transfer is the mainstay of reconstruction when the scalp is avulsed and cannot be replanted [11]. In our case, the avulsed scalp was crushed into pieces and pulled into the machine, making replantation impossible.

Free flap techniques ensure dependable wound closure and offer a range of reconstructive possibilities [8]. The latissimus dorsi muscle flap is commonly employed for large scalp reconstructions due to its size, reliable vascular pedicle, and effective results when covered with a STSG [13]. Only the muscle is harvested to avoid bulky subcutaneous fat, with skin grafting performed over it to maintain scalp contour as was done in our case [11]. The debate on single versus multiple vascular anastomosis in scalp replantation or free flap transfer is ongoing [14]. Our case involved a single vessel anastomosis between the thoracodorsal vessel pedicle and superficial temporal vessels [11].

Literature indicates high success rates and effectiveness of most flaps, even in elderly patients. However, cosmesis remains a significant concern with free flaps, particularly regarding issues like hair loss and discrepancies in color and shape [11].

In conclusion, scalp avulsions are frequent in workers exposed to machinery in factories and agricultural settings. Implementing occupational safety measures is crucial to reduce these injuries, especially in low- and middle-income countries. Flap coverage followed by graft is an effective and acceptable option when replantation is not feasible.

## Declaration

All the authors declare that the information provided here is accurate to the best of our knowledge.
